# Single Particle Tracking of α7 Nicotinic AChR in Hippocampal Neurons Reveals Regulated Confinement at Glutamatergic and GABAergic Perisynaptic Sites

**DOI:** 10.1371/journal.pone.0011507

**Published:** 2010-07-09

**Authors:** Thomas Bürli, Kristin Baer, Helge Ewers, Corinne Sidler, Christian Fuhrer, Jean-Marc Fritschy

**Affiliations:** 1 Institute of Pharmacology and Toxicology, University of Zurich, Zurich, Switzerland; 2 School of Medicine, Institute of Life Science, Swansea University, Swansea, United Kingdom; 3 Laboratory of Physical Chemistry, ETH Zurich, Zurich, Switzerland; 4 Department of Neurochemistry, Brain Research Institute, University of Zurich, Zurich, Switzerland; Vrije Universiteit Amsterdam, Netherlands

## Abstract

α7 neuronal nicotinic acetylcholine receptors (α7-nAChR) form Ca^2+^-permeable homopentameric channels modulating cortical network activity and cognitive processing. They are located pre- and postsynaptically and are highly abundant in hippocampal GABAergic interneurons. It is unclear how α7-nAChRs are positioned in specific membrane microdomains, particularly in cultured neurons which are devoid of cholinergic synapses. To address this issue, we monitored by single particle tracking the lateral mobility of individual α7-nAChRs labeled with α-bungarotoxin linked to quantum dots in live rat cultured hippocampal interneurons. Quantitative analysis revealed different modes of lateral diffusion of α7-nAChR dependent on their subcellular localization. Confined receptors were found in the immediate vicinity of glutamatergic and GABAergic postsynaptic densities, as well as in extrasynaptic clusters of α-bungarotoxin labeling on dendrites. α7-nAChRs avoided entering postsynaptic densities, but exhibited reduced mobility and long dwell times at perisynaptic locations, indicative of regulated confinement. Their diffusion coefficient was lower, on average, at glutamatergic than at GABAergic perisynaptic sites, suggesting differential, synapse-specific tethering mechanisms. Disruption of the cytoskeleton affected α7-nAChR mobility and cell surface expression, but not their ability to form clusters. Finally, using tetrodotoxin to silence network activity, as well as exposure to a selective α7-nAChR agonist or antagonist, we observed that α7-nAChRs cell surface dynamics is modulated by chronic changes in neuronal activity. Altogether, given their high Ca^2+^-permeability, our results suggest a possible role of α7-nAChR on interneurons for activating Ca^2+^-dependent signaling in the vicinity of GABAergic and glutamatergic synapses.

## Introduction

The α7-nicotinic acetylcholine receptor (α7-nAChR) differs amongst nAChRs by its homopentameric structure [1] and high calcium permeability [2], [3]. α7-nAChRs constitute high-affinity α-bungarotoxin (α-BT) binding sites in the CNS [4], [5]. They contribute to attention and memory [6], modulate cognitive functions [7], [8], and are considered a target for cognitive enhancers [9]. α7-nAChRs are most abundant in the hippocampus and neocortex, notably in GABAergic interneurons [10], where they mediate cholinergic synaptic input [11] and enhance GABAergic IPSCs in principal neurons [12]. Ultrastructural studies reported their presence predominantly at glutamatergic synapses on cortical pyramidal cells [13], [14]. α7-nAChRs are also located presynaptically, regulating release of various neurotransmitters [15], [16], [17], [18]. In primary hippocampal cultures, α7-nAChRs are prominent in interneurons, forming somato-dendritic clusters partially localized at GABAergic synapses [19]. We have confirmed these findings and demonstrated that α7-nAChR cell-surface distribution is regulated by interaction with PICK1 [20].

It is unclear, however, how α7-nAChR clusters are formed and positioned at specific somato-dendritic sites, notably because primary hippocampal neuron cultures are largely deprived of cholinergic synaptic input. This raises the general question of how α7-nAChRs lateral membrane diffusion is regulated. Formation of cell-surface receptor clusters can occur when stabilizing interactions affect lateral diffusion of single receptor molecules relative to adjacent membrane domains [21], [22]. In particular, interactions with scaffolding molecules underlies immobilization of some receptors in these clusters [22]. However, even though synaptic receptor clusters are stable over time, single particle tracking (SPT) studies revealed a dynamic equilibrium of individual molecules diffusing in and out of established clusters [23].

Functionally, mobility of neurotransmitter receptors contributes to regulate synaptic function by multiple mechanisms. For instance, since endocytosis occurs extrasynaptically [24], [25], [26], receptors have to diffuse out of the postsynaptic density (PSD) to be endocytosed. Therefore, receptor mobility modulates their cell surface expression [27]. Receptor mobility also might explain how desensitized receptors in postsynaptic sites are replaced within tens of milliseconds with non-desensitized receptors [28], and how differential dynamic fluctuations of synaptic and extrasynaptic receptors could account for plasticity of excitatory synapses [29]. In the case of α7-nAChRs, analyzing their lateral diffusion dynamics might help explaining the formation and function of cell-surface clusters at extrasynaptic and synaptic sites and identifying molecular interactions that underlie their transient immobilization.

This study addressed these issues by using fluorescence SPT [30] in living cultured hippocampal GABAergic interneurons to analyze the membrane dynamics of individual α7-nAChRs labeled with α-BT linked to quantum dots (QDs). Glutamatergic and GABAergic postsynaptic sites were visualized by recombinant expression of specific postsynaptic markers to determine the spatial distribution of α7-nAChR relative to these synapses. Since lateral diffusion of various ligand-gated ion channels is regulated by the cytoskeleton and synaptic activity [31], [32], [33], we tested whether α7-nAChR distribution and mobility are modulated by drugs disrupting tubulin or actin, and by neuronal firing or by activation or blockade of α7-nAChR.

## Materials and Methods

### Primary neuronal culture

All experiments were performed with primary rat hippocampal cell cultures prepared from E18 embryos taken from time-pregnant Wistar rats (RCC, Füllinsdorf, Switzerland), as previously described [34]. Cells were plated at a density of 40–50×10^3^ cells per 18 mm glass coverslip and cultured at 37°C/5% CO_2_ for about 3 weeks in minimal essential medium supplemented with 2% B27, 15 mM HEPES, 0.45% glucose monohydrate, 1 mM sodium pyruvate (all from Invitrogen, Basel, Switzerland), 2 mM L-glutamine (Gibco, Basel, Switzerland), 15% Nu-serum (Becton Dickinson, Basel, Switzerland). Experiments were done at 20–22 days *in vitro* (div).

### Transfection

Neuronal cultures were transiently transfected by magnetofection [34] with mCherry-Homer1c and EGFP-gephyrin. These proteins are selective markers of glutamatergic and GABAergic postsynaptic sites [35], [36]. After transfection in primary cultures, their postsynaptic localization in GABAergic interneurons was achieved upon long term expression. Thus, both constructs were transfected at 11 div; half of the medium was replaced with fresh medium to ensure cell growth until 20–22 div.

### Drug treatments

For pharmacological experiments different drugs were bath-applied into the culture medium for defined time periods before the experiments: biotinylated α-BT (Molecular Probes, Basel, Switzerland; 125 nM, dissolved in PBS); KCl (40 mM, dissolved in H_2_O); methyllycaconitine citrate hydrate (MLA), a selective α7-nAChR antagonist (Sigma-Aldrich, Buchs, Switzerland; 1 µM, dissolved in H_2_O); PNU-282987, a selective α7-nAChR agonist (Sigma-Aldrich; 300 nM, dissolved in DMSO); tetrodotoxin (TTX) (Sigma-Aldrich; 1 µM, dissolved in H_2_O), latrunculin A (Sigma-Aldrich; 3 µM, dissolved in DMSO) and nocodazole (Sigma-Aldrich; 10 µM, dissolved in DMSO), which depolymerise actin and tubulin, respectively.

### Immunocytochemistry

Post-hoc synapse labeling was performed after fixation of the cells for 10 min with 4% paraformaldehyde in 150 mM sodium phosphate buffer followed by permeabilisation for 5 min with 0.1% Triton® X-100, 10% normal goat serum (NGS; Serotec, Düsseldorf, Germany) in phosphate buffered saline (PBS). Primary rabbit polyclonal antibodies against vesicular glutamate transporter type 1 (vGluT1; Synaptic Systems, Göttingen, Germany, Nr 135 303; 1∶8000) and vesicular inhibitory amino acid transporter (VIAAT; Synaptic Systems, Nr 131 003; 1∶1000) were applied for 45 min in 10% NGS in PBS. Secondary goat anti-rabbit antibody coupled to Cy3 or Cy5 (Jackson ImmunoResearch, West Grove, PA; 1∶1000 and 1∶200) was incubated for 30 min in 10% NGS in PBS.

Images from transfected neurons were analyzed to determine the fraction of EGFP-gephyrin and mCherry-Homer1 clusters apposed to vGlut1 and VGAT-positive terminals, thereby ascertaining their postsynaptic location. Images were processed with a Gaussian filter [37] (ImageJ) to amplify small and large clusters with low intensities. Then, they were converted to 1-bit images with clusters having value 1 and background value 0. The relative number of EGFP-gephyrin and mCherry-Homer1 clusters and their mean surface area was then calculated in a sample of 7 cells from two independent cultures. Next, to quantify the apposition to presynaptic terminals, EGFP-gephyrin and mCherry-Homer1c clusters were enlarged by 1 pixel using a distance map filter (ImageJ), and colocalization with vGluT1 or VGAT immunofluorescence staining determined and counted using the “analyze particles tool” of ImageJ.

### Live staining

Surface α7-nAChRs on living cells were labeled with α-BT-AlexaFluor® 647 (α-BT AF647; Molecular Probes; 125 nM) for 2–5 min in cell-conditioned medium at 37°C/5% CO_2_.

Single receptor labeling of α7-nAChRs with QDs (QDOT®s; Invitrogen) was done at 4°C to reduce internalization and unspecific staining of QDs, as follows: Living cells were incubated with biotinylated α-BT (50–125 nM) in cell-conditioned medium for 5 min at 37°C/5% CO_2_. Cells were rinsed 3 times with 4°C PBS. Streptavidin-coupled QD605 or QD647 were preincubated at a concentration of 1–2 nM in freshly prepared 1% BSA Fraction V (Sigma-Aldrich) in PBS for 5 min at room temperature to avoid unspecific binding and were applied in a second staining round to the cells for 2 min at 4°C. Cells were rinsed subsequently 12 times in 4°C PBS containing 100 nM biotin to block remaining streptavidin-biotin binding sites. For live imaging, cells were covered with recording medium (minimum essential medium without phenol red supplemented with 15 mM HEPES, 0.45% glucose monohydrate, 1 mM sodium pyruvate, and 2 mM L-glutamine).

### Live microscopy and QD imaging

Live microscopy was performed on a Leica DMI6000b inverted microscope equipped with a 63× objective (NA 1.3). Cells were mounted in a metal chamber (Life Imaging Services, Basel, Switzerland) covered with recording medium, and kept at 5%CO_2_ and 37°C. Dyes were illuminated by a mercury lamp (EL6000; Leica). QD excitation and emission was controlled by specific filters (AHF, Tübingen, Germany). For detection an EM-CCD camera (C9100; Hamamatsu, Solothurn, Switzerland) was used (10^6^ pixels; pixel size, 0.125 µm×0.125 µm). Acquisition of images and movies was performed with the software Velocity (Improvision, Coventry, UK).

Prior to QD imaging, pictures of differential interference contrast (DIC), α-BT AF647, and transfected constructs were taken. Subsequently a movie of the QD labeled α7-nAChRs was recorded for 30–40 s with 50 ms exposure time at a rate of 20Hz. To limit phototoxicity cells were illuminated with the lowest possible light intensity and sessions lasted maximally 45 min.

### Single particle tracking and analysis

For better handling, recorded movies were converted from RAW to AVI format using ImageJ (Rasband, 1997–2008). Single QDs were identified by their on-off blinking behavior. Trajectories of single QDs were tracked by using the ImageJ plugin Particle Tracker [38]. Trajectory interruptions due to off phases of the QDs were interpolated and subtrajectories were linked if the phase did not exceed more than 10 consecutive frames. The maximal displacement of a QD from frame to frame was set to 3 pixels (375 nm). Trajectories consisting of less than 100 frames were excluded from further analysis.

Trajectory analysis was done with custom software written in Excel Visual Basic. The mean square displacement (MSD) of a trajectory was calculated according to the following equation [39]: 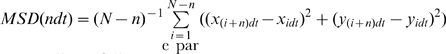 where *x*(*i*) and *y*(*i*) is the particle position of a trajectory with *N* frames at frame *i* with a frame time interval of *dt*. By fitting the first 5 data points of the MSD versus time (*t*) curve the diffusion coefficients (*D*) was calculated with the equation: 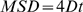. Instantaneous diffusion coefficients were derived accordingly over contiguous trajectory stretches of 20 frames.

To distinguish between random movements and confined behavior of receptors diffusing within a limited area, the confinement index L was calculated as described [40], [41]. L is a probability level reflecting the tendency for non-random confinement. When averaged over time, it can be used as a confinement index. As shown in the references above [40], [41], L>3.16 for 2.5 sec has a likelihood of >99.3% to reflect confined mobility. This threshold was used here for identifying episodes of confined diffusion. Finally, the confinement surface area L^2^ was estimated by fitting the MSD plot with the following equation [33], [42]: 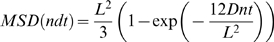


To determine at any time point of its trajectory the distance of a QD to the nearest postsynaptic site, clusters of EGFP-gephyrin or mCherry-Homer1c were binarized as described above, and the distance to the nearest cluster was determined at every position of the image by applying a distance map filter (ImageJ). From these data, the distribution of instantaneous locations as a function of a distance from the PSD was calculated. This distribution was normalized to the surface area occupied by image pixels around PSDs. The PSD was considered as being circular with a mean radius of 0.1 µm (as determined from their average size). The surface area of concentric circles around the PSD, spaced by 0.125 µm (pixel size), was then used for calculating the number of subtrajectories present around the PSD as a function of distance.

Next, we arbitrarily defined EGFP-gephyrin and mCherry-Homer1 clusters as representing a synaptic zone, and, based on the distribution curves of the instantaneous location of QDs, a radius of 4 pixels (<500 nm) around these clusters as representing a perisynaptic zone. Membrane areas further than 500 nm from the edge of clusters were defined as extrasynaptic.

Measuring the distance of QDs from clusters thus allowed to split and classify trajectories into synaptic, perisynaptic and extrasynaptic parts, and to determine the instantaneous diffusion coefficient and dwell time in these compartments. Calculation of distance from a cluster vs. diffusion coefficient and confinement vs. diffusion coefficient was enabled by taking the instantaneous diffusion coefficient into account.

### Data and statistical analysis

Data are presented as mean±SEM. Statistical analyses were done using Prism® 4 (GraphPad Software, La Jolla, CA) and SPSS 11.5 (SPSS Inc., Zürich, Switzerland).

## Results

### α7-nAChRs aggregate in perisynaptic clusters

**Figure 1:**
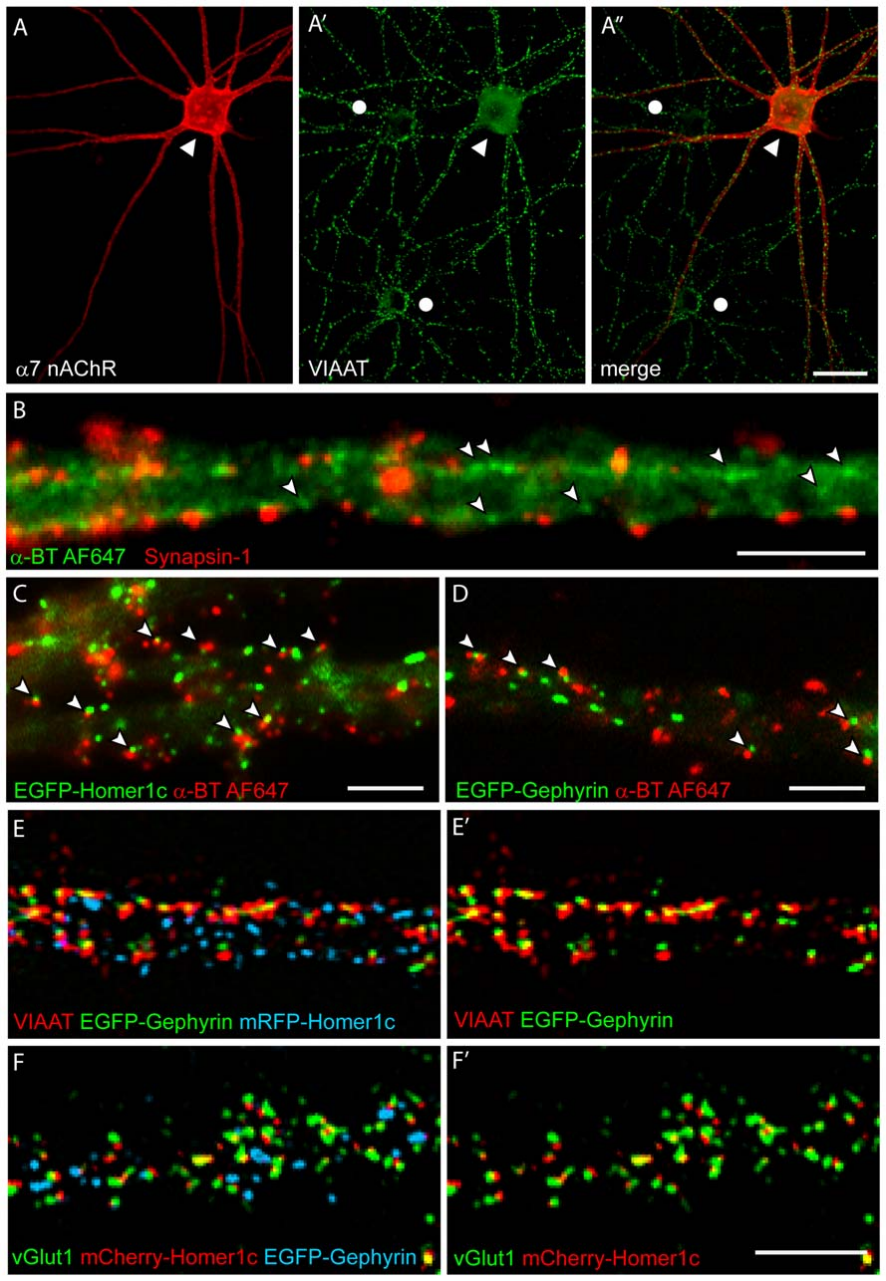
Characterization of interneurons used for SPT of α7-nAChRs at 21 div. (A) Fluorescent α-BT (red) selectively labels α7-nAChRs in interneurons positive for vesicular inhibitory amino acid transporter (VIAAT;▴). Neighboring neurons show no somatic VIAAT staining (•) but are surrounded by GABAergic synapses. (B) Fluorescent α-BT clusters are opposed to the presynaptic marker Synapsin-1, but larger clusters are presumably extrasynaptic (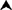). (C–D) Predominant perisynaptic localization of fluorescent α-BT clusters, as shown by their apposition (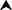) to mCherry-Homer1c and EGFP-gephyrin clusters in living interneurons transfected with one of these markers. (E–F) Segregation of mCherry-Homer1c and EGFP-gephyrin (co-transfected by magnetofection at 11 div) between excitatory and inhibitory postsynaptic sites, as shown by immunofluorescence staining for VIAAT (E–E′) and vGluT1 (F–F′) at 21 div. Note the selective opposing of the labeled terminals to the corresponding postsynaptic marker. Image was enlarged to depict the pixel array detected by the CCD camera. Scale bars: A, 40 µm; B–F, 5 µm.

**Figure 2:**
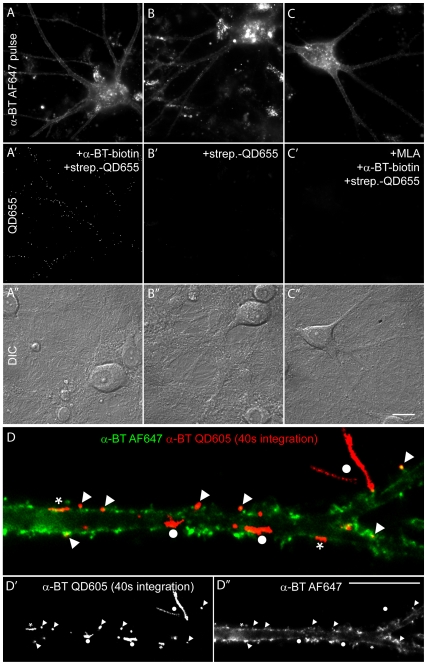
Selectivity of single α7-nAChR labeling with streptavidin-QD bound to biotinylated α-BT. (A–C) α7-nAChR-positive hippocampal neurons were identified by a pulse staining with fluorescent α-BT (α-BT AF647). Single α7-nAChRs were labeled with biotinylated α-BT followed by streptavidin-QD655 (A′); omission of α-BT (B′) or previous blocking of α7-nAChR with 1µM methyllycaconitine (MLA) for 15 min (C′) abolished QD binding. (A″–C″) Differential interference contrast (DIC) images of the same field of view illustrate the extensive neuronal network around the labeled cells. (D–D″) Single α7-nAChRs were immobilized in α-BT-positive clusters. Cell-surface α7-nAChRs were labeled at t = 0 sec with α-BT AF647 (green). The 40 s trajectories of single QD (red traces) revealed different modes of motion, including QDs confined in strongly stained α-BT clusters (▴); short trajectories in moderately stained α-BT clusters; 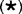; and long trajectories outside α-BT clusters (•). The corresponding single labeled images are depicted in D′ and D″. Scale bars: C″ (for panels A–C), 20 µm; D″, 10 µm.

In rat primary hippocampal neurons cultured for 21 div, prominent α-BT labeling of α7-nAChRs was detected selectively on GABAergic interneurons identified by VIAAT-immunofluorescence (Fig. 1A). As described previously [19], α-BT labeling was not uniform but revealed individual clusters on the soma and dendrites of positive neurons (Fig. 1B; Fig. 2). To determine whether these clusters were located at or in the vicinity of synapses, we combined α-BT labeling with immunofluorescence of synapsin-1, a marker present in most synapses in primary hippocampal cultures. The majority of α-BT-positive clusters was located extrasynaptically (1.8±0.1% α7-nAChR clusters were colocalized with synapsin-1; 19.8±0.5% were apposed to synapsin-1 terminals, and 78.4±0.5% were independent of them; n = 11 cells) (Fig. 1B).

To better understand the localization of α-BT clusters in relation to post-synaptic sites, we co-transfected cultured rat hippocampal neurons at 11 div with plasmids encoding fluorescently tagged recombinant markers of GABAergic (EGFP-gephyrin, [43]) and glutamatergic (mCherry-Homer1c, [44]) postsynaptic sites, and investigated their distribution in α7-nAChR-positive cells at 21 div. This approach was necessary because the intricate networks of neurites formed in the dish and the limited resolution of immunofluorescence make it almost impossible to unequivocally attribute immuno-labeled synapses to a given cell.

A substantial fraction of α-BT-positive clusters were localized at close proximity to either glutamatergic or GABAergic postsynaptic sites (Fig. 1C–D). They typically were apposed to, rather than co-localized with, clusters of Homer1c and gephyrin, suggesting a perisynaptic localization of α7-nAChRs Thus, 1.7±0.2% of α7-nAChR clusters colocalized with gephyrin clusters, whereas 14.3±0.7% were apposed to gephyrin clusters (n = 6 cells; Student's t test; p<10^−3^); 1.9±0.2% of α7-nAChR clusters colocalized with Homer1c clusters, whereas 17.4±1.0% were apposed to Homer1c clusters (n = 6 cells; Student's t test; p<10^−3^). Since α7-nAChRs are also present in presynaptic terminals *in vivo*, notably at excitatory synapses, one might argue that their “perisynaptic” localization seen by fluorescence microscopy reflects accumulation in presynaptic terminals. However, this interpretation is unlikely because such perisynaptic clusters were only seen in interneuron-like cells strongly labeled by α-BT over their entire soma and dendritic tree, as illustrated in Figure 1A and Figure 2. Therefore, these experiments indicate a preferential perisynaptic localization of α7-nAChRs in hippocampal interneurons

In control experiments, the correct localization of transfected gephyrin and Homer1c in relation to presynaptic input was verified by immunofluorescence staining for VIAAT and vGlut1 (Fig. 1E–F; Suppl. Fig. S1). Both recombinant proteins were co-expressed in most transfected neurons, including interneurons. After 21 div, they were expressed at moderate levels and were detectable mainly as brightly stained clusters that were distinctly segregated along dendrites, likely representing sites of synaptic input. The average size of these clusters, as measured on a random sample of 500 clusters each from 7 cells after selection with a Gaussian filter (see Materials and Methods), was 0.061±0.034 µm^2^ (radius, 0.14 µm) for mCherry-Homer1c and 0.126±0.047 µm^2^ (radius, 0.2 µm) for EGFP-gephyrin. Clusters smaller than 2 pixels or larger than 1 µm^2^ were excluded from this quantification. It is of note that interneurons typically are not spiny and receive both glutamatergic and GABAergic synapses on their shaft [45]. Furthermore, in double labeling experiments, 46.7±6.3% (SEM) of the gephyrin clusters and 39.0±6.2% (SEM) of the Homer1c clusters were opposed to VIAAT- and vGlut1-positive terminals, respectively. Mismatched synapses were present but in a significantly smaller proportion than matched synapses (22.7±4.4% gephyrin clusters apposed to vGlut1; 16.5±3.5% Homer1c clusters apposed to VIAAT; n_vGlut1_ = 6 cells; p = 0.04; n_VIAAT_ = 5 cells; p_VIAAT/Gephyrin/Homer1c_ = 0.003; Student's t tests). Therefore, these markers allowed distinguishing glutamatergic from GABAergic synapses formed onto identified transfected interneurons. The relatively high proportion on clusters not apposed to a synaptic marker (31.6% EGFP-gephyrin and 44.5% mCherry-Homer1c) is unlikely due to an overexpression artifact, because the majority of these isolated clusters was present in fine dendritic branches, not innervated by labeled axon terminals. In addition, glutamatergic containing vGluT2 have not been considered.

### α7-nAChR mobility is reduced in α7-nAChR clusters

The lateral mobility of α7-nAChRs in hippocampal interneurons was analyzed by SPT, with the aim to determine whether specific synaptic and extrasynaptic domains regulate their diffusion kinetics and therefore their subcellular distribution. Single α7-nAChRs were tagged in living cells with biotinylated α-BT and, in a second step, labeled with streptavidin-QDs. Only a small fraction of α7-nAChRs were bound to α-BT, avoiding chronic blockade of cholinergic activation. Since QDs have the tendency to bind non-specifically in primary neuronal cultures, the staining procedure was tested extensively for specificity and optimized to minimize non-specific labeling (Fig. 2A–C). Thus, to identify α7-nAChR-positive cells within the dense neuronal network, a minority of α7-nAChR was first pulse-labeled with α-BT coupled to the fluorochrome AF647 (Fig. 2A). The subsequent QD labeling of single α7-nAChR overlapped perfectly with the α-BT AF647 fluorescence (Fig. 2A′), indicating that non-specific binding of QDs was negligible. Furthermore, labeling was abolished by prior application of 1 µM MLA, a selective α7-nAChR antagonist [46] (Fig. 2C′). Finally, no signal was seen when streptavidin-QDs were applied in the absence of α-BT (Fig. 2B′), confirming the specificity of our protocol.

To analyze the mobility of α7-nAChRs, the movements of single QD labeled receptors in living cells were recorded for 40 s with a frame rate of 20 Hz (Fig. 2D–D″; supplementary Movie S1). Labeling of single α7-nAChR was ascertained by the blinking behavior of QDs. Frames of the recorded movies were merged yielding a single image showing the trajectories of single α7-nAChRs (Fig. 2D). These trajectories were overlaid onto a still image displaying the overall distribution of α7-nAChRs labeled with fluorescent α-BT at t = 0 s (Fig. 2D′). These overlays revealed the presence of free, as well as confined, QDs in areas with different content of α7-nAChRs (Fig. 2D″). Per definition, a confined receptor remains within an area of the membrane for a longer time than predicted from Brownian diffusion. As evident in the supplementary Movie S1, very short trajectories were made by nearly immobile QDs; however, they were not always colocalized with strong α-BT labeling (Fig. 2D). Intermediate trajectories, measuring 3–5 µm, typically were apparent by highly mobile QDs in zones of weak α-BT labeling, whereas QDs on unlabeled filopodia or axons displayed apparently unconfined mobility within the limits of theses neurites. These observations suggest that areas with high α7-nAChR content correspond mainly to membrane domains where α7-nAChR mobility is low and where some α7-nAChRs are transiently confined by an unknown mechanism.

### α7-nAChRs exhibit different modes of motion

The proximity of α7-nAChR clusters to excitatory and inhibitory synapses suggest that α7-nAChR might be confined perisynaptically. To test this hypothesis, single receptors were tracked in interneurons co-transfected with EGFP-gephyrin and mCherry-Homer1c. Their trajectories were analyzed quantitatively to determine their mean square displacement (MSD), diffusion coefficient (D), confinement index (L), and confinement area L^2^ (see Materials and Methods). The MSD is an indicator of how freely a molecule can move. A linear increase over time indicates free diffusion, whereas an asymptotic or flat MSD time-curve is characteristic for confined motion. D describes the instantaneous mobility of a moving particle, but provides no information about the restriction of diffusion. L, averaged over time, allows the identification of periods of confined diffusion.

**Figure 3:**
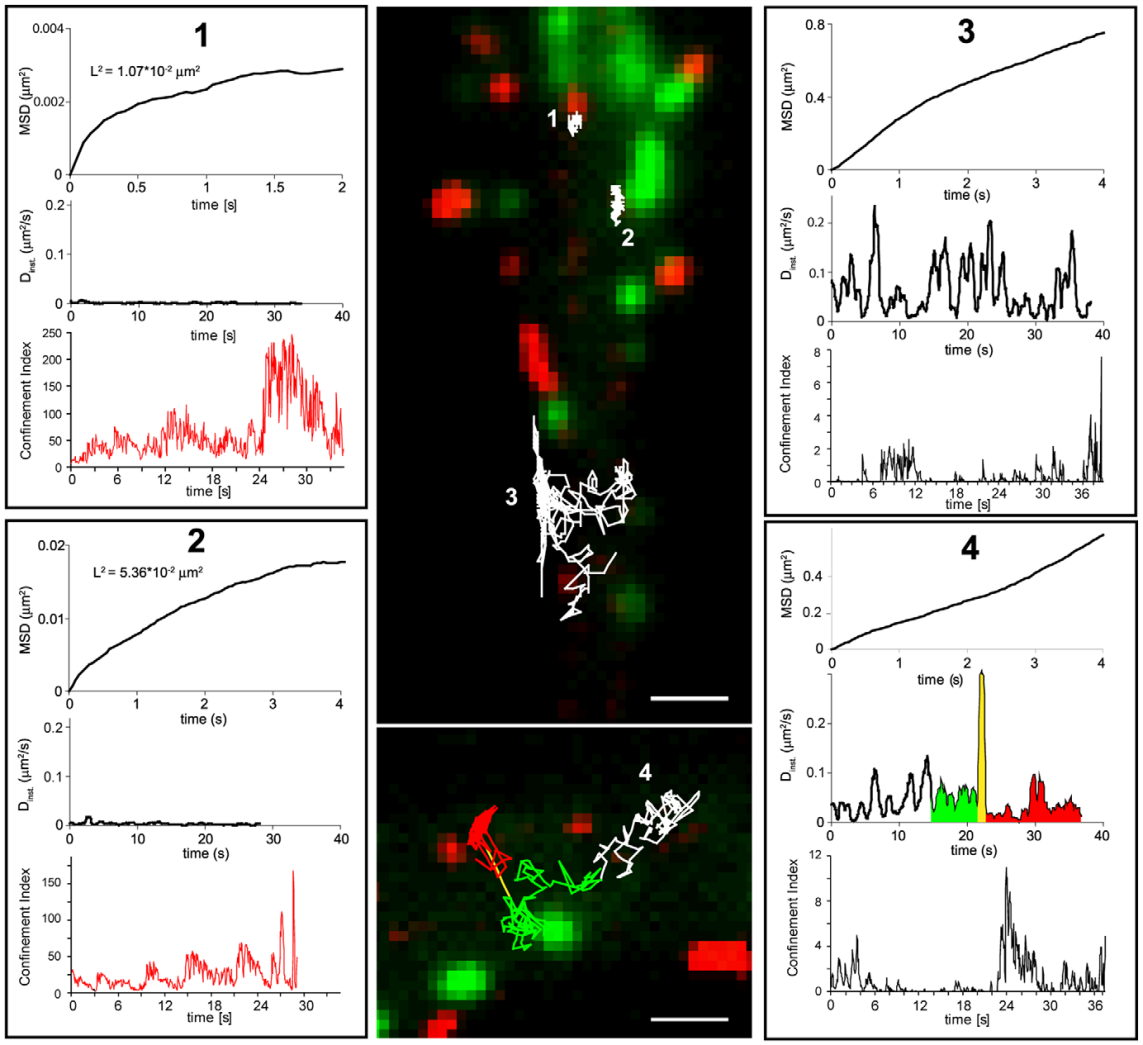
Various diffusive behaviors of single QD-labeled α7-nAChRs. The trajectories of single QDs (shown in white) were recorded over 40 s in 21 div hippocampal interneurons transfected at 11 div with EGFP-gephyrin (green) and mCherry-Homer1c (red) (middle). (1–4) Diagrams of mean square displacement (MSD, top), instantaneous diffusion coefficient (D, middle), and confinement index (L, bottom) of single quantum dots as a function of their location (indicated in the middle panel). A red trace indicates confined mobility, as determined by the L index over time. (1,2) Asymptotic MSD, small D and small confinement surface area L^2^ values reflect strong confinement of α7-nAChR at excitatory and inhibitory perisynaptic localizations, respectively; note that the trajectories do not enter the PSD in these two examples. (3) Free diffusion of a QD located in an extrasynaptic domain. (4) Example of a QD swapping between EGFP-gephyrin and mCherry-Homer1c clusters, with reduced D in proximity to the respective clusters; the various parts of the trajectory are color-coded. This QD does not remain confined during the recorded time. Scale bar: 1 µm.

MSD, D, L, and L^2^ were calculated for all trajectories longer than 100 successive frames (5 s at 20 Hz). In total, 21 interneurons from 3 independent transfection experiments were examined, with 30–40 QDs being recorded per neuron. The trajectories of single α7-nAChRs could be coarsely categorized as illustrated in Figure 3. Confined α7-nAChR, with small asymptotic MSD, D close to 0, large L, and small L^2^ were detected in close vicinity of mCherry-Homer1c and EGFP-gephyrin clusters (Fig. 3; examples 1 and 2). Such receptors typically were apparently excluded from the PSD; rather, they were located perisynaptically within 1–4 pixels of the margin of the Homer1c or gephyrin cluster. In addition to perisynaptic sites, confinement of α7-nAChR was also observed at extrasynaptic sites (supplementary Movie S2), suggesting the existence of subdomains in which MSD is low, likely corresponding to the extrasynaptic clusters seen with fluorescent α-BT (Fig. 1B). Nevertheless, most extrasynaptic QDs were highly mobile, exhibiting MSD increasing linearly over time, and D values of ∼0.05–0.1 µm^2^/s, reaching maxima ∼0.3 µm^2^/s (Fig. 3; example 3); accordingly, the trajectories of such QDs were indicative of random lateral diffusion in the dendritic membrane. A small number of QDs reached even higher mobility with D>0.3 µm^2^/s (supplementary Movie S2). α7-nAChRs with such high D occurred in structures where diffusion practically is reduced to one dimension, such as axons or filopodia. Finally, a fourth mode of motion discovered was the one of “swapping” QDs. These α7-nAChRs were extrasynaptic but moved towards both inhibitory and excitatory perisynaptic sites, in which they remained for several seconds, albeit without necessarily being confined (Fig. 3; example 4; supplementary Movie S2).

Dwell times at perisynaptic sites were variable, often lasting several seconds. Long-time recordings even revealed α7-nAChR displaying dwell times of more than 40 min (data not shown). Altogether, the various trajectories illustrated in Fig. 3 confirm that α7-nAChRs are highly mobile extrasynaptically but are slowed down or even confined at excitatory and inhibitory perisynaptic sites, as well as in certain extrasynaptic domains.

### α7-nAChR mobility differs in excitatory and inhibitory perisynaptic sites

**Figure 4:**
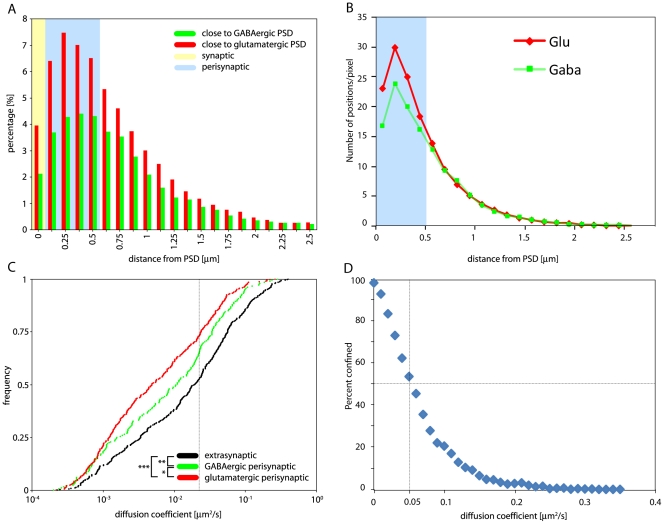
Confinement of α7-nAChRs at perisynaptic sites. (A) Distribution of instantaneous position of single QD-labeled α7-nAChR as a function of the distance to the nearest postsynaptic site. Each subtrajectory was assigned to either of two groups, depending on the closest proximity of a mCherry-Homer1c or EGFP-gephyrin cluster. The distribution reveals selective accumulation close to Homer1c- and gephyrin-positive PSDs. The entire range of distances measured is shown (sum of all data points = 100%). α7-nAChRs were more frequently localized in proximity to Homer1c cluster compared to EGFP-gephyrin clusters and avoided entering the PSD (n_glutamatergic_ = 2564, n_GABAergic_ = 1687). Perisynaptic domains (blue background) were defined as corresponding <500 nm from the nearest PSD (yellow background). (B) Data from panel A were normalized to the number of instantaneous positions of QDs per pixel area (125 nm×125 nm) in function of the distance from the nearest PSD. These data were calculated from the surface area of virtual rings around each PSD, with an average diameter of 200 nm. Note the exponential decline, with the half-maximal value reached at 0.5 mm from the edge to the PSD (4th ring), thereby defining the outer border of the “perisynaptic” area (blue). (C) Cumulative frequency distribution of D of α-BT-labeled QDs in perisynaptic and extrasynaptic subtrajectories α7-nAChRs around mCherry-Homer1c and EGFP-gephyrin-positive clusters, showing a significant difference between glutamatergic and GABAergic perisynaptic domains (n_glutamatergic_ = 358, n_GABAergic_ = 206, n_extrasynaptic_ = 369; Kolmogorov-Smirnoff; p_GABAergic/glutamatergic_ = 0.022, p_glutamatergic/extrasynaptic_<10^−3^, p_GABAergic/extrasynaptic_ = 0.007); the dotted line indicates the fraction of QD in each membrane domain having a D<0.05 µm^2^. (D) Fraction of QDs exhibiting confined mobility (as determined by calculating the L index, see Materials and Methods) as a function of their D; the dotted lines indicate that 50% of QDs with confined mobility have a D<0.05; the corresponding proportion of QDs are depicted in panel C.

The confinement of α7-nAChRs was prominent perisynaptically, but not within postsynaptic sites. To further analyze this finding, the trajectories of the 777 QDs recorded in interneurons co-transfected with EGFP-gephyrin and mCherry-Homer1c were investigated more closely. First, we determined the instantaneous localization of each QD with respect to presumptive PSD labeled with these markers. In every frame of a given trajectory, we assigned the QD to one of two groups, depending on whether it was located closer to a Homer1c or to a gephyrin cluster. Next, the distance to the closest cluster was determined and the instantaneous diffusion coefficient D calculated. The distribution of QDs as a function of their distance to the nearest postsynaptic cluster, is shown in Figure 4A, distinguishing between the two groups (glutamatergic and GABAergic postsynaptic clusters). Defining 0 nm as the edge of the PSD, this histogram shows that α7-nAChRs had a bell shaped distribution, being most frequently located close to glutamatergic or GABAergic PSDs. This analysis also confirmed our visual impressions that QDs avoid entering postsynaptic sites (Supplementary Movie S2), as only 2% and 4% of the recorded instantaneous positions of QDs were localized over a gephyrin or a Homer1c cluster, respectively. It also showed that α7-nAChRs are more frequent in vicinity of Homer1c clusters (27.5% of all instantaneous positions) than gephyrin clusters (16%) (Fig. 4A). This difference still held true upon normalization of the number of excitatory and inhibitory PSD on interneuron dendrites (Supplementary Fig. S2A). The preferential localization of QDs around postsynaptic sites was confirmed taking into account the relative surface area covered by concentric imaginary rings surrounding every postsynaptic cluster. The number of instantaneous locations per pixel declined exponentially with distance from both GABAergic and glutamatergic PSDs (Fig. 4B). Based on this distribution, we set an arbitrary virtual boundary between a perisynaptic and an extrasynaptic domain at the half maximal density of positions/pixel, representing a distance of 4 pixels (0.5 µm) from the edge of the PSD (Fig. 4B).

To determine whether this preferential perisynaptic distribution of α7-nAChRs was due to changes in mobility as a function of localization, instantaneous D of individual QDs were calculated as function of the distance to the nearest PSD. This analysis showed that the instantaneous mobility of QDs was highly variable within either postsynaptic, perisynaptic, or extrasynaptic domains, with no significant differences among the three domains (Supplementary Fig. S2B). Therefore, the formation of perisynaptic α7-nAChR aggregates cannot be explained solely by decreased instantaneous D, arguing for specific perisynaptic tethering mechanisms that retain receptors around PSDs.

To explain why more α7-nAChRs were seen in the vicinity of glutamatergic synapses than GABAergic synapses, we next analyzed the average mobility of QDs within each of the three compartments defined above (extrasynaptic, perisynaptic-Homer1c, perisynaptic-gephyrin). To this end, trajectories of individual QDs were split into subtrajectories when they crossed the border between two compartments and their average D within each subtrajectory were calculated. Analysis of these data revealed that subtrajectories of perisynaptic QDs assumed a broad range of D, in line with their highly variable instantaneous mobility, as shown on a cumulative frequency distribution plot (Fig. 4C). Statistical analysis showed that D in subtrajectories of single QDs around Homer1c- and gephyrin-positive clusters were significantly different from each other and from extrasynaptic QDs (Kolmogorov-Smirnoff; p_GABAergic/glutamatergic_ = 0.022, p_glutamatergic/extrasynaptic_<10^−3^, p_GABAergic/extrasynaptic_ = 0.007). Accordingly, the average values for D_glutamatergic_ = (0.018±0.03µm^2^/s), D_GABAergic_ (0.028±0.04 µm^2^/s), and D_extrasynaptic_ (0.046±0.07 µm^2^/s) were significantly different (One-way ANOVA; F2,843 = 23.82; p<0.001). This result suggested that the tethering mechanisms holding α7-nAChRs around glutamatergic and GABAergic PSDs are different.

Finally, examination of the diffusion coefficient data of all subtrajectories of QDs undergoing random and confined diffusion revealed that more than 50% of QD with a D<0.05 µm^2^/s were confined; conversely, D>0.1 µm^2^/s were typical of non-confined mobility (Fig. 4D). Since more than 66% “GABAergic” and 70% “glutamatergic” perisynaptic subtrajectories had a D<0.05 µm^2^/s (Fig. 4C), α7-nAChRs with low mobility were confined within perisynaptic domains adjacent to Homer1c and gephyrin clusters. This observation confirms that perisynaptic domains are endowed with specific mechanisms to retain a subset of receptors.

### α7-nAChR cluster maintenance is independent of an intact actin cytoskeleton and functional microtubules

**Figure 5:**
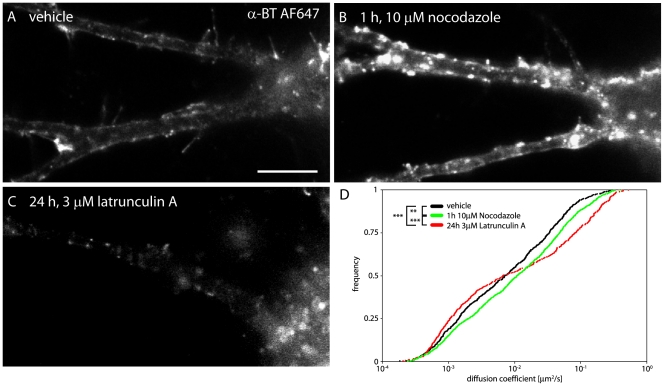
Effect of latrunculin A and nocodazole on α7-nAChR clustering and mobility. (A) Hippocampal neurons were treated with vehicle, (B) 10µM nocodazole for 1h, (C) and 3µM latrunculin A for 24 h to interfere with the actin cytoskeleton and the microtubule network, respectively. Each panel shows a picture of α-BT AF647 labeling. Loss of filamentous actin caused reduced surface staining while the loss of microtubules caused an increase of surface α7-nAChRs (B, C). However, both treatments did not prevent α-BT clustering. (D) Analysis of α7-nAChR mobility revealed a significant increase upon disruption of the microtubule network. The depolymerization of the actin network led to removal of intermediate fast receptors whilst slow and fast receptors persisted (n_vehicle_ = 735, n_nocodazole_ = 943, n_latrunculin A_ = 407; Kolmogorov-Smirnoff; p_vehicle/nocodazole_ = 0.006, p_vehicle/latrunculin A_<10^−3^, p_nocodazole/latrunculin A_<10^−3^). Scale bar: 10 µm.

To uncover such a retention mechanism stabilizing α7-nAChRs, and also to understand how stable, apparently “extrasynaptic” clusters are maintained in dendrites, we tested whether the tubulin or actin cytoskeleton are involved. These experiments were conducted using non-transfected cultures, in which a higher number of interneurons are present for analysis. The sample analyzed was derived from two independent culture batches and includes 12 cells treated with vehicle (DMSO), 23 cells treated with latrunculin A, and 9 cells treated with nocodazole. A high number of cells treated with latrunculin was necessary to obtain sufficient QD trajectories for quantitative analysis (>400 per condition). Microtubule disruption by nocodazole (1h, 10 µM; [33]) markedly increased the surface α-BT fluorescence compared to vehicle-treated cultures (Fig. 5A–B), possibly due to reduced turnover of cell-surface α7-nAChRs. This effect did not affect formation of α7-nAChR clusters, despite significantly increased D of single QDs (Fig. 5D; Kolmogorov-Smirnoff; p_vehicle/nocodazole_ = 0.006). Complete depolymerization of actin microfilaments requires prolonged latrunculin A exposure (24 h, 3 µM) [47]. This treatment led to a pronounced reduction of α-BT fluorescence labeling. Analysis of the D of remaining QDs revealed increased fractions of confined/slowly diffusing receptors and of highly mobile/non-confined receptors at the expense of QDs with “intermediate” mobility, corresponding to extrasynaptic α7-nAChRs (example 3 in Fig. 3) (Kolmogorov-Smirnoff; p_vehicle/latrunculin A_<10^−3^, p_nocodazole/latrunculin A_<10^−3^). Therefore, latrunculin A treatment might increase endocytosis of extrasynaptic receptors on dendrites, but does not directly disrupt pre-established α7-nAChR clusters. Collectively, these experiments indicate that formation and maintenance of α7-nAChR clusters occurs independently of the cytoskeleton, suggesting that they are linked to protein networks within the membrane.

### Synaptic activity affects α7-nAChR mobility

**Figure 6:**
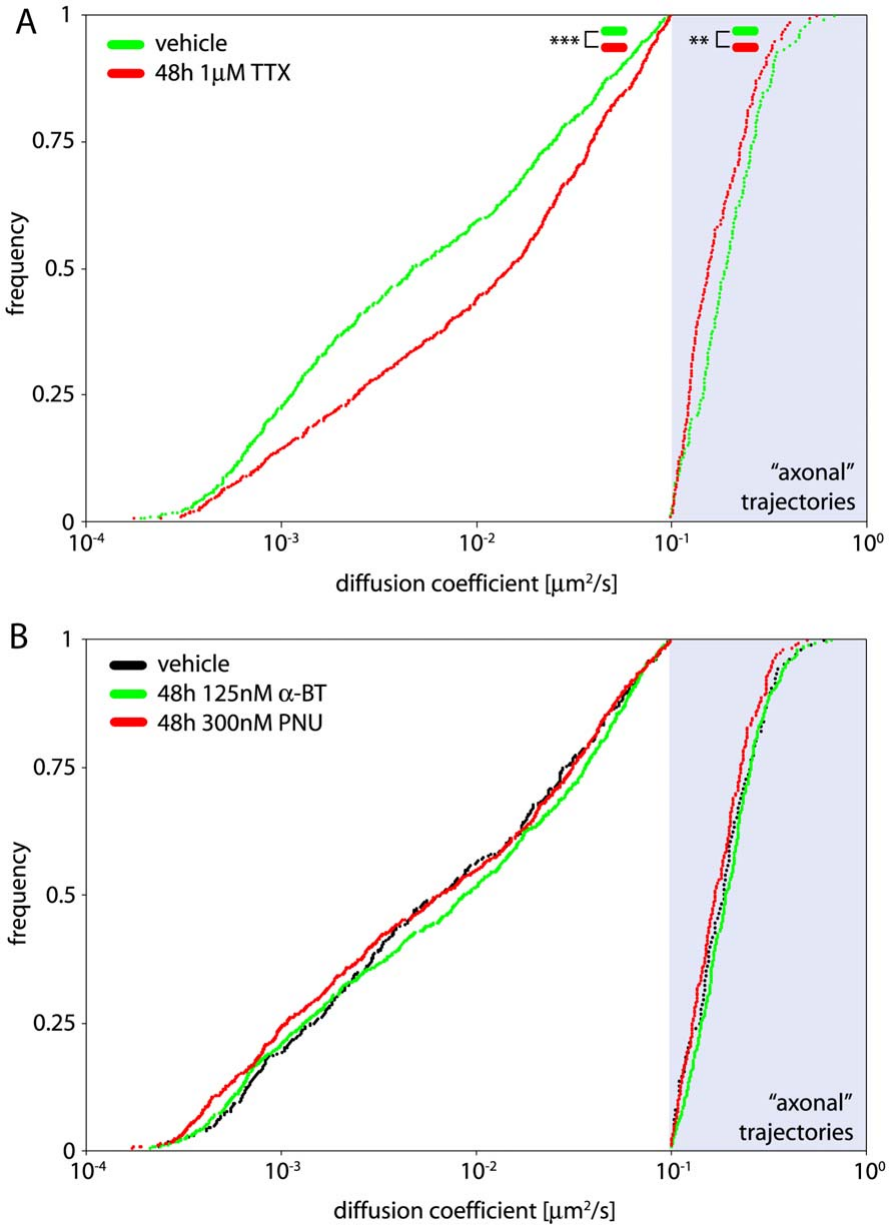
Activity-dependent regulation of cell surface mobility of α7-nAChRs. Trajectories with D>0.1µm^2^/s were assumed to be axonal. (A) Neuronal firing influenced mobility of α7-nAChRs. Blockade of voltage gated sodium channels with 1 µM tetrodotoxin (TTX) for 48 h in 21 div hippocampal neurons increased α7-nAChRs mobility (n_vehicle(“non-axonal”)_ = 636, n_TTX(“non-axonal”)_ = 718, n_vehicle(“axonal”)_ = 136, n_TTX(“axonal”)_ = 155; Kolmogorov-Smirnoff; p_”non-axonal”_<10^−3^, p_”axonal”_ = 0.007). (B) Chronic application of the α7-nAChR agonist PNU-282987 (300 nM) or the antagonist α-BT-treated (100 nM, 48 h) for 48 h to 21 div hippocampal neurons causes no significant effect on α7-nAChR mobility (n_vehicle(“axonal”)_ = 106, n_α-BT(“axonal”)_ = 298, n_PNU(“axonal”)_ = 203).

The subcellular localization and aggregation of ligand-gated ion channels is regulated by synaptic activity [31], [32]. We tested several reagents applied for variable time periods on 21 div hippocampal neurons for their effect on α7-nAChR surface mobility. As in the previous section, these experiments were conducted using non-transfected cultures, precluding the analysis of QD mobility at identified GABAergic or glutamatergic perisynaptic sites. For quantification, “dendritic” and “axonal” α7-nAChRs were discriminated according to their diffusion coefficient. Based on visual observations of trajectories of highly mobile QDs, “axonal” α7-nAChRs, which account for ∼20% of all α7-nAChRs, were found to exhibit diffusion coefficients >0.1µm^2^/s suggesting that they undergo Brownian motion. The threshold to distinguish these from “non-axonal” receptors was arbitrarily set at 0.1 µm^2^/s. In a first series of experiments performed on 6 cells each from two independent cultures, acute treatment with KCl (40 mM, 20 s), TTX (1 µM, 30 min), and PNU-282987 (α7-nAChR agonist; 300 nM, 30 min) had no effect on α7-nAChR mobility during this time, as determined by cumulative probability analysis of diffusion coefficients of the same receptors before and after drug treatment (not shown). Prolonging the KCl treatment has deleterious effects on cell morphology, precluding analysis. A second series of experiments performed with cells chronically exposed to TTX at 21 div (48 h; 1 µM; n = 11 and 13 for vehicle treatment, taken from two independent cell culture batches) revealed strongly reduced cell surface expression of α7-nAChRs (not shown), as published previously [19]. This effect was accompanied by a highly significant increase in mobility of α7-nAChRs residing on dendrites (from 0.018±0.024 to 0.025±0.027 µm^2^/s; t = 5.015 df = 1352, p = 0.0023) whereas axonal QDs exhibited a moderate reduction in mobility (from 0.218±0.104 to 0.187±0.04 µm^2^/s; t = 2.811 df = 289, p = 0.0103) (Fig. 6A). These observations suggest that prolonged silencing of neuronal activity leads to increased mobility of α7-nAChRs, thereby favoring their internalization by endocytosis, as seen upon disruption of actin microfilaments.

Finally, we tested whether chronic α7-nAChR activation or blockade had an effect on their own diffusion behavior (Fig. 6B). In parallel experiments, we tested on 21 div neurons the effect of 48 h exposure of α7-nAChR to 125 nM biotinylated α-BT and 300 nM PNU-282987, respectively (n = 6 for vehicle, 18 for α-BT, and 9 for PNU-282987, taken from three independent cell culture batches). These treatments produced non-significant difference in the mobility of QDs compared to vehicle (Fig. 6B). Taken together, we conclude that overall synaptic activity, but not chronic activation or blockade of α7-nAChRs, regulates cell surface expression and mobility of α7-nAChRs.

## Discussion

The present results show that α7-nAChRs are aggregated either extrasynaptically or in close vicinity to both glutamatergic and GABAergic synapses in cultured hippocampal interneurons. Analysis of their membrane dynamics by SPT revealed different modes of lateral diffusion dependent on their location and on interactions with the cytoskeleton. The lowest receptor mobility, reflecting local confinement domains, coincided with perisynaptic sites around Homer1c and gephyrin clusters, as well as in extrasynaptic clusters of α-BT labeling on dendrites. α7-nAChRs avoided entering PSDs, but exhibited confined mobility and long dwell times in glutamatergic and GABAergic perisynaptic sites, suggesting differential, synapse-specific tethering mechanisms. These findings suggest a role for α7-nAChR in modulating Ca^2+^-activated signaling cascades at GABAergic and glutamatergic synapses on interneurons. Finally, the mobility of α7-nAChRs was regulated by global network activity, with chronic application of TTX resulting in increased mobility and reduced cell surface expression. These combined effects suggest reduced α7-nAChRs confinement in silenced neurons, leading to internalization of receptors diffusing laterally in the plasma membrane.

### Localization and SPT of α7-nAChRs

α-BT labeling confirmed that α7-nAChRs form prominent clusters on interneurons *in vitro* [19], with a heterogeneous distribution in relation to synaptic sites. Thus, synapsin-1 labeling distinctly revealed large clusters present at extrasynaptic sites, whereas co-transfection of EGFP-Homer1c and mCherry-gephyrin indicated that “synaptic” α7-nAChRs clusters rather are located perisynaptically in the close vicinity of both glutamatergic and GABAergic PSDs. Based on these observations, we analyzed the lateral mobility of α7-nAChRs to understand the mechanisms underlying this heterogeneous distribution. Due to the extrinsic nature of cholinergic input to the hippocampus, α7-nAChR in cultured hippocampal neurons are not associated with cholinergic synapses. Therefore, several additional aspects of our study differ from previous SPT investigations of ligand-gated ion channels, which deserve consideration:

A major potential confounding factor was that some α7-nAChRs are presynaptic on axon terminals (see Introduction). We used co-expression of mCherry-Homer1c and EGFP-gephyrin to distinguish glutamatergic and GABAergic postsynaptic sites and ensure that all markers analyzed were present in the same cell. However, long-term expression was required until recombinant proteins formed clusters distinctly apposed to glutamatergic and GABAergic axon terminals on interneurons. The cell culture and transfection conditions had to be optimized to achieve these results [34], with the advantage that α7-nAChRs were investigated in mature neurons having stable synaptic contacts.

A major issue related to the identification of postsynaptic sites, as well as perisynaptic domains in which QDs exhibit confined mobility, is whether the resolution of fluorescence microscopy is sufficient to draw conclusions about the location of QDs in these two compartments. In a previous study, analysis of the diffusion behavior of QDs labeled with antibodies to GlyRs revealed different modes of motion within gephyrin clusters and in perisynaptic domains in spinal cord neurons [48]. Here, using a similar approach, we observed a different distribution pattern of QDs labeled with α-BT, which were confined outside of PSDs labeled with mCherry-Homer1c or with EGFP-gephyrin, being located within a narrow margin from the edge of these PSDs. These consistent observations strongly suggests that image resolution was not a limiting factor biasing our conclusions. Therefore, the apparent perisynaptic localization of α7-nAchRs likely reflects the existence of tethering mechanisms holding these receptors in the membrane around synaptic sites. However, quantitative analysis of the distribution of QDs around postsynaptic sites revealed exponential decay as a function of distance rather than a sharp border. This distribution curve likely reflects uncertainty of the location of QDs due to the resolution of fluorescence imaging.

Here, α7-nAChRs were specifically labeled with biotinylated α-BT and streptavidin-coated QDs. Compared to latex beads coupled to receptors [49] or immunocytochemistry to link the QD to the receptor [23], α-BT is therefore favorable for SPT, because its small size reduces the risk of mobility hindrance. Extensive investigations by other groups revealed that QDs do not alter receptor mobility, which is determined by the physico-chemical properties of the plasma membrane [50]. Only in protein-dense environments, such as the synaptic cleft, a reduction in the freedom of mobility has been observed. However, since receptors linked to QDs with IgGs have been shown to diffuse into PSDs, it is unlikely that the small fraction of “synaptic” QDs observed in this study reflects this limitation.

SPT analysis required labeling of a small fraction of α7-nAChR and minimal background arising from non-specific streptavidin binding. To fulfill these requirements and avoid receptor cross-linking, which affects their diffusion coefficient [28], as well as functional inactivation, we used a low concentration of α-BT and blocked exposed biotin binding sites on streptavidin-QD with free biotin immediately after the labeling procedure.

At the concentration used here, α-BT-binding sites were prominent selectively in VIAAT-positive cells. Accordingly, the majority of QDs analyzed by SPT were located on dendrites. Nevertheless, given the extensive axonal network formed in 21 div cultures, the presence of axonal α7-nAChRs was to be expected. They were recognized by their high diffusion coefficient along narrow, straight trajectories, which were clearly distinct from the trajectories of dendritic QDs, representing postsynaptic α7-nAChRs. Based on this distinction, “axonal” QDs were arbitrarily defined for quantitative analysis of their mobility as having a D >0.1 µm^2^/s.

Finally, the excellent concordance between fluorescent α-BT labeling and SPT trajectories on labeled cells confirmed random QD labeling across the entire population of α7-nAChRs; consequently, the vast majority of trajectories (∼80%) analyzed here correspond to dendritic receptors.

### Diffusion behavior of α7-nAChR

Multi-molecule approaches in living cells, such as FRAP or cell surface labeling with pH-sensitive GFP, have provided considerable insight into the mechanisms regulating membrane protein accumulation at specific pre- and postsynaptic domains [21]. However, since the site of membrane insertion (or removal) is often distant from the site of function, the lateral mobility of single receptor molecules must be taken into account to understand how their synaptic aggregation and function are regulated. Thus, SPT experiments uncovered fast AMPAR recruitment to active synapses by lateral diffusion from extrasynaptic pools [31] and showed a rapid diffusive exchange of desensitized receptors with non-desensitized receptors [28]. Most importantly, SPT studies demonstrated that receptor clusters are stable entities despite the fact that single receptors constantly diffuse in and out and exchange between clusters [51].

Our analysis of α7-nAChR cell surface mobility shows that single receptors display a broad range of diffusion coefficients similar to other ligand-gated ion channels [23], [27]. As expected, their confinement coincided with clusters of α-BT labeling. Some receptors swapped between clusters, showing free diffusion behavior in extrasynaptic areas and reduced mobility after entering glutamatergic or GABAergic perisynaptic sites. Interestingly, confinement was inversely correlated with mobility, as reported for GlyR stabilized in gephyrin clusters [48]. However, while GlyRs could be subdivided between “immobilized” receptors and “swapping” receptors entering gephyrin clusters just for short dwell times, no comparable separation was apparent here for α7-nAChRs, which were seen only rarely in the PSD marked by EGFP-gephyrin.

A major finding is the observation that α7-nAChR diffusion coefficients and distribution were different between glutamatergic and GABAergic perisynaptic sites. While their dwell times were similar in both perisynaptic areas, their diffusion coefficient was significantly smaller in glutamatergic sites. Further, a larger fraction of perisynaptic α7-nAChR was located at glutamatergic sites, suggesting more efficient trapping than at GABAergic sites. We have shown that PICK1 interacts with α7-nAChR and regulates its clustering [20]. PICK1 is enriched in glutamatergic postsynaptic sites, regulating AMPAR trafficking and synaptic transmission [52]. Long-term overexpression of PICK1 (or the inactive PDZ-domain mutant) was attempted but resulted in toxicity; SPT analysis after 24 h revealed no significant effect of PICK1 on α7-nAChR mobility (not shown). Additional experiments to downregulate PICK1 by RNA silencing also were unsuccessful in our hands.

Aggregation of receptors within the cell membrane occurs through stabilization and binding to other proteins [47]. In particular, receptors can be linked via intermediate proteins to actin microfilaments or to microtubules. AMPAR, for instance, are stabilized by GRIP1, which binds to microfilaments [47]. In contrast, GlyR are stabilized extrasynaptically by microtubuli and synaptically by gephyrin and actin filaments [33]. Receptors can also be stabilized by a highly cross-linked network of membrane proteins, without requiring cytoskeletal elements [53]. This might be the case for α7-nAChRs, since α7-nAChR clusters were not disrupted upon depolymerization of actin filaments and microtubules, whereas α7 nAChR cell surface expression and mobility changed significantly. In ciliary ganglion neurons, α7-nAChRs clusters were identified in lipid rafts [54]. Here, staining of lipid rafts using cholera toxin subunit B revealed no colocalization with α-BT clusters in hippocampal neurons (unpublished data). Therefore, the mechanisms underlying stabilization of α7-nAChR in specific membrane subdomains remain to be determined.

### Modulation of α7-nAChR mobility

GlyR cell surface mobility is altered within seconds of NMDAR activation [32]. In contrast, 48 h blockade of synaptic activity with TTX was required to decrease AMPAR diffusion [29]. Rapid effects on receptor mobility presumably reflect posttranslational or conformational changes, whereas delayed effects might require activation of gene expression. We observed here that acute induction or repression of synaptic activity by KCl and TTX, respectively, or exposure to the α7-nAChR agonist PNU-282987, had no significant effect on α7-nAChR mobility. In contrast, chronic TTX treatment for 48h resulted in an extensive loss of α7-nAChR clusters in interneurons as previously described [19], accompanied by a significant increase in receptor mobility. This finding suggests that the loss of α7-nAChR is due to receptor cluster dispersion and subsequent internalization.

### Role of perisynaptic α7-nAChRs

Our study uncovers differential, activity-dependent α7-nAChR lateral mobility and perisynaptic confinement by selective tethering mechanisms, suggesting a role for regulating glutamatergic and GABAergic synaptic function through Ca^2+^-mediated mechanisms. Thus, in hippocampal interneurons, stimulation of α7-nAChRs profoundly depresses GABAergic IPSCs as well as muscimol-evoked GABAergic currents in a Ca^++^- and PKC-dependent manner, indicating a postsynaptic modulation of GABA_A_ receptors [55]. At glutamatergic synapses α7-nAChRs modulate back-propagating dendritic action potentials and contribute to long-term plasticity of interneurons [56]. Likewise, neuronal depolarization through nAChR helps to relieve the Mg^2+^ block of postsynaptic NMDAR, thereby enhancing the probability of LTP induction [57]. Studies at GABAergic synapses in chick ciliary ganglion and rodent hippocampus showed that α7-nAChRs downregulate GABA-induced currents by a CaMKII- and MAPK-dependent mechanism [58]. More generally, Ca^2+^ influx might be necessary to induce LTP at GABAergic synapses [59] and Ca^2+^-dependent activation of nNOS is required to maintain presynaptic LTP_GABA_ [60]. Therefore, owing to their high Ca^2+^ permeability, perisynaptic α7-nAChRs are located in a strategic position to activate intracellular signaling pathways regulating GABAergic and glutamatergic synapses independently of their endogenous transmitter.

## Supporting Information

Figure S1 Segregated distribution of mCherry-Homer1c (red) and EGFP-gephyrin (green) in interneurons co-transfected by magnetofection at 11 div and processed for immunofluorescence staining for vGluT1 (A) or VIAAT (B) (blue) at 21 div. The boxed areas are enlarged panels A′ and B′. Arrowheads point to postsynaptic clusters apposed to a labeled presynaptic terminal. Arrows indicate isolated, possibly non-synaptic clusters. Scale bars, 20 µm.
(4.90 MB TIF)

Figure S2 A) Normalized distribution of single QD-labeled α7-nAChR as a function of the distance to the nearest postsynaptic site. The distribution was calculated upon normalization of the ratio of mCherry-Homer1c and EGFP-gephyrin clusters on interneuron dendrites. These data confirm that α7-nAChR trajectories are more frequently localized in glutamatergic than in GABAergic perisynaptic sites (Fig. 5A). B) Distribution of the dwell time of single QD-labeled α7-nAChRs as a function of their diffusion coefficient in perisynaptic sites. The negative correlation (r2GABAergic = 0.91, r2glutamatergic = 0.87) between both parameters indicates that α7-nAChRs with a small diffusion coefficient tend to stay longer in perisynaptic sites (nglutamatergic = 2106; nGABAergic = 1445).
(0.47 MB TIF)

Movie S1 Single α7-nAChRs are immobilized in α-BT-positive clusters. Cell-surface α7-nAChRs were labeled at t = 0 s with α-BT AF647 (green). Single QD labeled α7-nAChRs (red) reveal different modes of motion, including QDs confined in strongly stained α-BT clusters, slow but mobile QDs in moderately stained α-BT clusters, and fast mobile QDs outside α-BT clusters. Scale bar: 10 µm.
(11.34 MB AVI)

Movie S2 Single QD-labeled α7-nAChRs (white) in 21 div hippocampal interneurons transfected at 11 div with EGFP-gephyrin (green) and mCherry-Homer1c (red). Receptors are showing various different behaviors. Extrasynaptic confined and mobile, perisynaptic confined in glutamatergic and GABAergic synaptic sites and receptors swapping between perisynaptic areas. Scale bar: 1 µm.
(11.71 MB AVI)
