# Supplementary figures and images for: Single Particle Tracking of α7 Nicotinic AChR in Hippocampal Neurons Reveals Regulated Confinement at Glutamatergic and GABAergic Perisynaptic Sites
Source: PLoS One. 2010 Jul 9;5(7):e11507. doi: 10.1371/journal.pone.0011507 (PMC2901346; doi:10.1371/journal.pone.0011507)

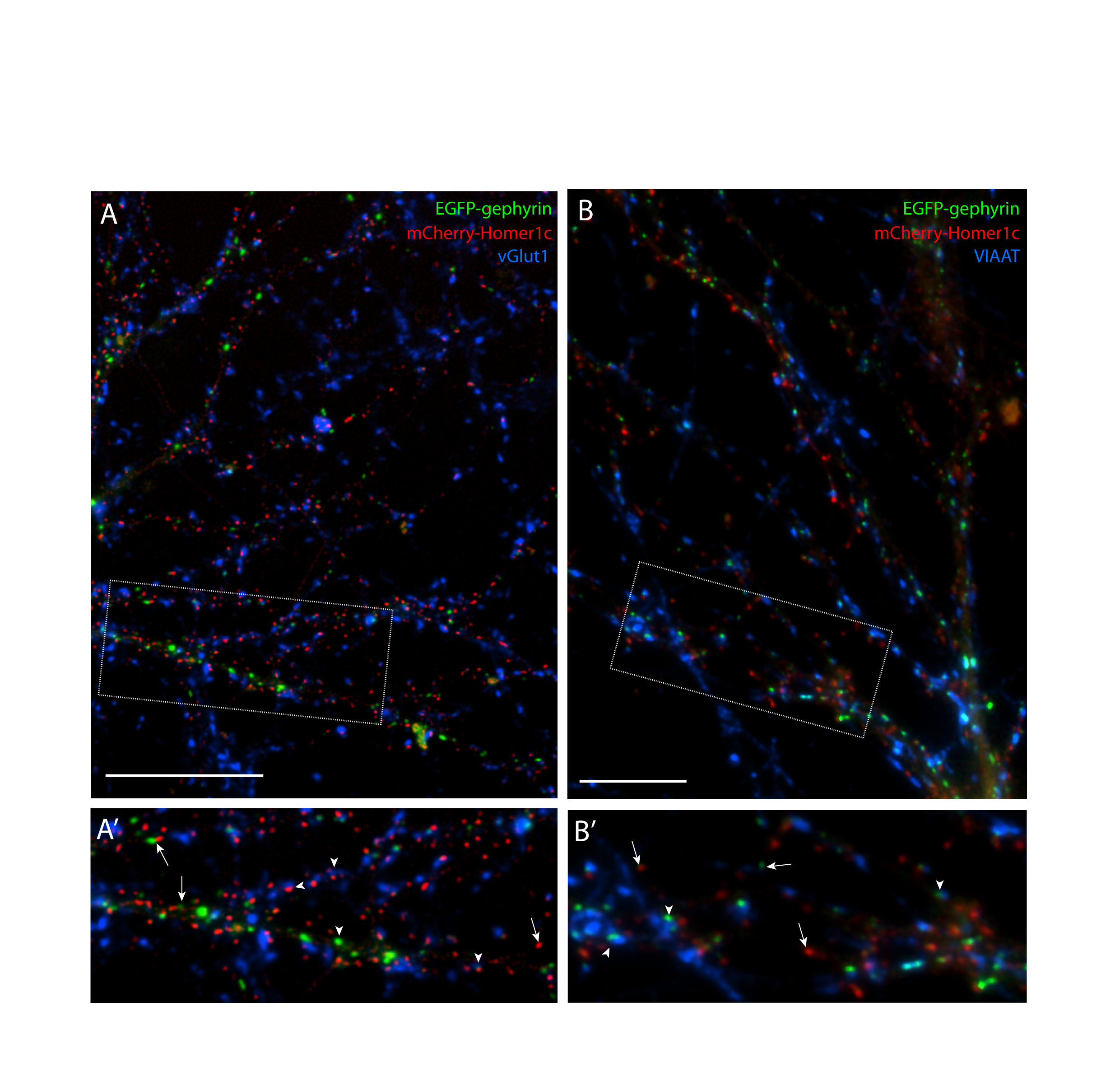

Supplement: Figure S1 — Segregated distribution of mCherry-Homer1c (red) and EGFP-gephyrin (green) in interneurons co-transfected by magnetofection at 11 div and processed for immunofluorescence staining for vGluT1 (A) or VIAAT (B) (blue) at 21 div. The boxed areas are enlarged panels A′ and B′. Arrowheads point to postsynaptic clusters apposed to a labeled presynaptic terminal. Arrows indicate isolated, possibly non-synaptic clusters. Scale bars, 20 µm. (4.90 MB TIF) [file pone.0011507.s001.tif]

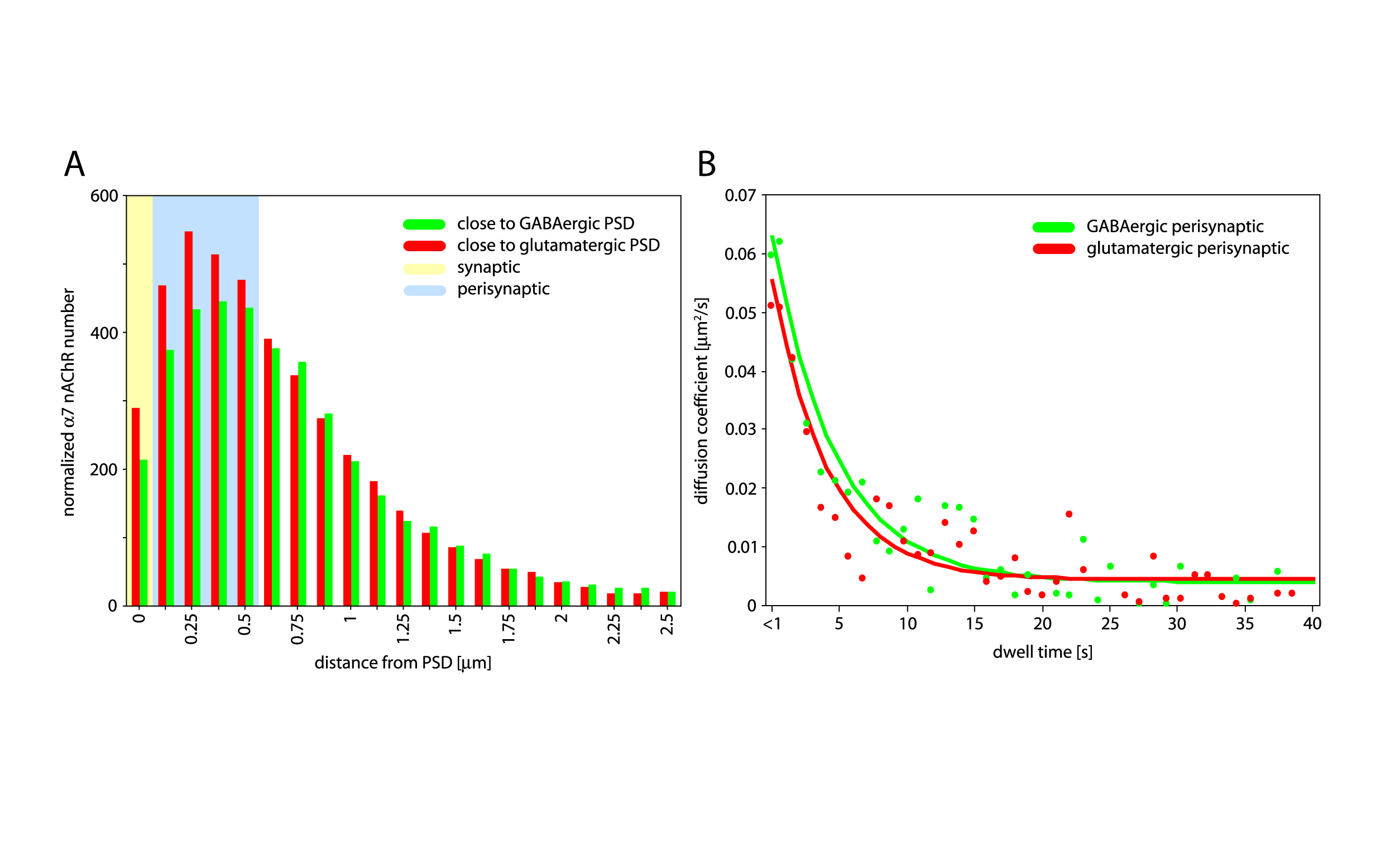

Supplement: Figure S2 — A) Normalized distribution of single QD-labeled α7-nAChR as a function of the distance to the nearest postsynaptic site. The distribution was calculated upon normalization of the ratio of mCherry-Homer1c and EGFP-gephyrin clusters on interneuron dendrites. These data confirm that α7-nAChR trajectories are more frequently localized in glutamatergic than in GABAergic perisynaptic sites (Fig. 5A). B) Distribution of the dwell time of single QD-labeled α7-nAChRs as a function of their diffusion coefficient in perisynaptic sites. The negative correlation (r2GABAergic = 0.91, r2glutamatergic = 0.87) between both parameters indicates that α7-nAChRs with a small diffusion coefficient tend to stay longer in perisynaptic sites (nglutamatergic = 2106; nGABAergic = 1445). (0.47 MB TIF) [file pone.0011507.s002.tif]
